# Elranatamab in relapsed or refractory multiple myeloma: phase 2 MagnetisMM-3 trial results

**DOI:** 10.1038/s41591-023-02528-9

**Published:** 2023-08-15

**Authors:** Alexander M. Lesokhin, Michael H. Tomasson, Bertrand Arnulf, Nizar J. Bahlis, H. Miles Prince, Ruben Niesvizky, Paula Rodrίguez-Otero, Joaquin Martinez-Lopez, Guenther Koehne, Cyrille Touzeau, Yogesh Jethava, Hang Quach, Julien Depaus, Hisayuki Yokoyama, Afshin Eli Gabayan, Don A. Stevens, Ajay K. Nooka, Salomon Manier, Noopur Raje, Shinsuke Iida, Marc-Steffen Raab, Emma Searle, Eric Leip, Sharon T. Sullivan, Umberto Conte, Mohamed Elmeliegy, Akos Czibere, Andrea Viqueira, Mohamad Mohty

**Affiliations:** 1 Division of Hematology and Oncology, Memorial Sloan Kettering Cancer Center/Weill Cornell Medical College, New York City, NY USA; 2 Department of Internal Medicine, University of Iowa, Iowa City, IA USA; 3 Hôpital Saint-Louis, Paris, France; 4 Arnie Charbonneau Cancer Institute, University of Calgary, Calgary, Alberta Canada; 5 Epworth Healthcare and University of Melbourne, Melbourne, Victoria Australia; 6 Weill Cornell Medical College/New York Presbyterian Hospital, New York City, NY USA; 7 Clinica Universidad de Navarra, Madrid, Spain; 8 Hospital Universitario 12 de Octubre, Madrid, Spain; 9 Miami Cancer Institute, Miami, FL USA; 10 University Hospital of Nantes, Nantes, France; 11 Indiana Blood & Marrow Transplant, Indianapolis, IN USA; 12 University of Melbourne, St. Vincent’s Hospital Melbourne, Melbourne, Victoria Australia; 13 Université Catholique de Louvain, CHU UCL Namur, Yvoir, Belgium; 14 Tohoku University Graduate School of Medicine, Sendai, Japan; 15 Beverly Hills Cancer Center, Beverly Hills, CA USA; 16 Norton Cancer Center, Louisville, KY USA; 17 Winship Cancer Institute, Atlanta, GA USA; 18 Lille University Hospital and INSERM UMR-S1277, Lille, France; 19 Massachusetts General Hospital Cancer Center, Harvard Medical School, Boston, MA USA; 20 Department of Hematology & Oncology, Nagoya City University Graduate School of Medical Sciences, Nagoya, Japan; 21 Heidelberg Myeloma Center, Department of Hematology/Oncology, Heidelberg University Hospital, Heidelberg, Germany; 22 The Christie Hospital, The University of Manchester, Manchester, UK; 23 Pfizer Inc, Cambridge, MA USA; 24 Pfizer Inc, New York, NY USA; 25 Pfizer Inc, San Diego, CA USA; 26 Pfizer SLU, Madrid, Spain; 27 Sorbonne University, Hôpital Saint-Antoine, and INSERM UMRs938, Paris, France

**Keywords:** Myeloma, Drug development, Myeloma, Cancer immunotherapy

## Abstract

Elranatamab is a humanized B-cell maturation antigen (BCMA)-CD3 bispecific antibody. In the ongoing phase 2 MagnetisMM-3 trial, patients with relapsed or refractory multiple myeloma received subcutaneous elranatamab once weekly after two step-up priming doses. After six cycles, persistent responders switched to biweekly dosing. Results from cohort A, which enrolled patients without prior BCMA-directed therapy (*n* = 123) are reported. The primary endpoint of confirmed objective response rate (ORR) by blinded independent central review was met with an ORR of 61.0% (75/123); 35.0% ≥complete response. Fifty responders switched to biweekly dosing, and 40 (80.0%) improved or maintained their response for ≥6 months. With a median follow-up of 14.7 months, median duration of response, progression-free survival and overall survival (secondary endpoints) have not been reached. Fifteen-month rates were 71.5%, 50.9% and 56.7%, respectively. Common adverse events (any grade; grade 3–4) included infections (69.9%, 39.8%), cytokine release syndrome (57.7%, 0%), anemia (48.8%, 37.4%), and neutropenia (48.8%, 48.8%). With biweekly dosing, grade 3–4 adverse events decreased from 58.6% to 46.6%. Elranatamab induced deep and durable responses with a manageable safety profile. Switching to biweekly dosing may improve long-term safety without compromising efficacy. ClinicalTrials.gov identifier: NCT04649359.

## Abstract (web summary)

In a pivotal phase 2 trial, elranatamab, a bispecific antibody targeting B-cell maturation antigen (BCMA) and CD3, had a confirmed objective response rate of 61% in patients with relapsed or refractory multiple myeloma who had not previously received BCMA-directed therapy.

## Main

The introduction of immunomodulatory drugs, proteasome inhibitors and anti-CD38 monoclonal antibodies has transformed the treatment landscape in multiple myeloma. The addition of these agents has substantially improved patient survival; however, outcomes for patients with disease progression after these agents remain poor with a median progression-free survival (PFS) of 4.6 months and median overall survival (OS) of 12.4 months with a standard of care therapy, highlighting an unmet medical need in the relapsed or refractory multiple myeloma population^1^. In recent years, the development of T-cell-redirecting therapies has shown promise in this patient population^2^.

B-cell maturation antigen (BCMA), a member of the tumor necrosis factor receptor superfamily, is highly expressed on malignant plasma cells, making it an ideal target for the treatment of multiple myeloma^3^. A number of BCMA-directed therapies, including belantamab mafodotin, idecabtagene vicleucel (ide-cel), ciltacabtagene autoleucel (cilta-cel) and teclistamab, have shown efficacy in clinical trials and are approved for the treatment of relapsed or refractory multiple myeloma^4–9^.

Elranatamab (PF-06863135) is a humanized bispecific antibody that targets both BCMA (on myeloma cells) and CD3 (on T cells)^3^. Elranatamab activates and directs T cells to induce a cytotoxic T-cell response against myeloma cells^10^. Preliminary data from the ongoing phase 1 MagnetisMM-1 study (NCT03269136) demonstrated encouraging safety and efficacy of elranatamab in patients with relapsed or refractory multiple myeloma^11–14^.

The registrational phase 2 MagnetisMM-3 study (NCT04649359) evaluated the efficacy and safety of elranatamab monotherapy in patients with relapsed or refractory multiple myeloma^15,16^. Results in patients without prior BCMA-targeted treatment (cohort A) after ~15 months of follow-up, including clinical experience in patients who switched to biweekly dosing after persistent response, are reported. Cohort B, which enrolled patients previously treated with BCMA-directed therapies, will be reported separately.

## Results

### Trial design and patients

**Fig. 1:**
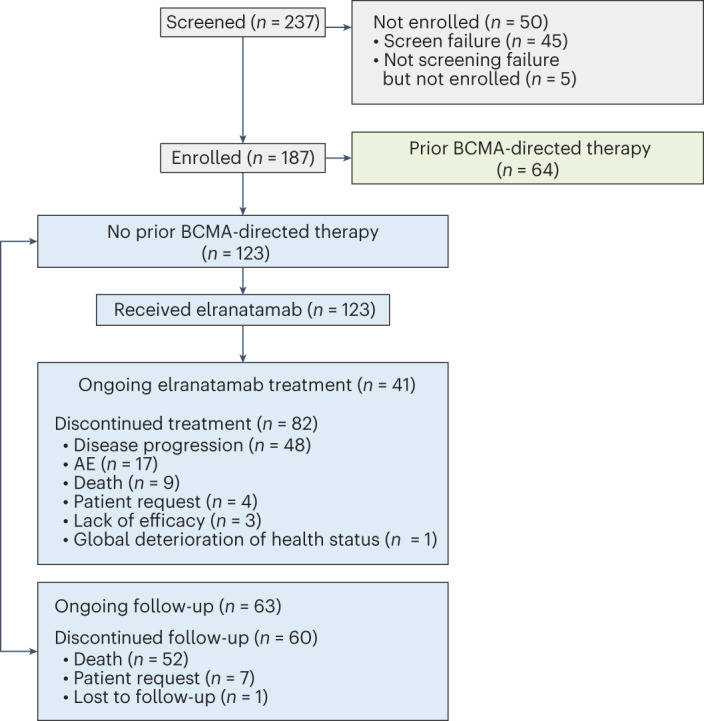
**CONSORT diagram of MagnetisMM-3.**

MagnetisMM-3 is an ongoing, multicenter, open-label, single-arm, phase 2 study investigating the efficacy and safety of elranatamab in patients with relapsed or refractory multiple myeloma. Eligible patients were 18 years of age or older with a prior diagnosis of multiple myeloma and measurable disease per International Myeloma Working Group (IMWG) criteria, adequate bone marrow (platelets ≥25 × 10^9^ l^−1^, absolute neutrophil count ≥1.0 × 10^9^ l^−1^, hemoglobin ≥8 g dl^−1^), hepatic (total bilirubin ≤2× upper limit of normal (ULN; ≤3x ULN if documented Gilbert’s syndrome), aspartate aminotransferase ≤2.5× ULN and ≤2.5× ULN alanine aminotransferase) and renal (creatinine clearance ≥30 ml min^−1^) function, and an Eastern Cooperative Oncology Group (ECOG) performance status ≤2. Patients had to have disease refractory to at least one proteasome inhibitor, one immunomodulatory drug and one anti-CD38 antibody, and disease relapsed or refractory to their last antimyeloma regimen. Those in cohort A must not have received prior BCMA-directed therapy. From February 9, 2021, to January 7, 2022, a total of 123 patients were enrolled in cohort A and dosed at 47 study sites in ten countries (Fig. 1 and Supplementary Table 1).

The primary endpoint was objective response rate (ORR) by blinded independent central review (BICR) per IMWG criteria^17^. Secondary endpoints included ORR by BICR baseline extramedullary disease status, ORR by investigator, complete response (CR) rate (defined as CR or better), time to response (TTR), duration of response (DOR), duration of CR or better (DOCR), minimal residual disease (MRD) negativity rate, PFS, OS, safety, pharmacokinetics and immunogenicity. Adverse events (AEs) and laboratory abnormalities were graded by National Cancer Institute Common Terminology Criteria for Adverse Events (NCI CTCAE) v5.0, and CRS and immune effector cell-associated neurotoxicity syndrome (ICANS) were graded according to the American Society for Transplantation and Cellular Therapy criteria^18^. Patients received subcutaneous elranatamab 76 mg once weekly in 28-d cycles after two step-up priming doses of 12 mg and 32 mg given on day 1 and day 4 of cycle 1. After six cycles, persistent responders (partial response (PR) or better lasting at least 2 months) switched to a dosing interval of once every 2 weeks (Q2W).

Among the 123 patients who received elranatamab, the median age was 68 years (range: 36–89 years), 55.3% were male, 58.5% were White, 13.0% were Asian and 7.3% were Black/African American (Table 1). At baseline, 63.4% of patients had an ECOG performance status of 1 or 2, 15.4% had stage III disease according to the Revised International Staging System (R-ISS) and 25.2% had high-risk cytogenetics, defined as t(4;14), t(14;16) or del(17p). Extramedullary disease, defined as the presence of any plasmacytoma (extramedullary and/or paramedullary with a soft-tissue component), assessed by BICR, was present in 31.7% of patients. Overall, 76.4% had at least one poor prognostic feature (Table 1). Patients had received a median of 5 (range: 2–22) prior lines of therapy, 96.7% had triple-class refractory disease and 42.3% had penta-drug refractory disease (refractory to at least two proteasome inhibitors, two immunomodulatory drugs and one anti-CD38 antibody).

**Table 1:** Baseline characteristics and prior treatment

Characteristics	Total (*n* = 123)
Median age (range), years	68.0 (36–89)
Male, *n* (%)	68 (55.3)
Race, *n* (%)	
White	72 (58.5)
Asian	16 (13.0)
Black or African American	9 (7.3)
Not reported or unknown^a^	26 (21.1)
Geographical region, *n* (%)	
North America	58 (47.2)
Europe	45 (36.6)
Asia	12 (9.8)
Other	8 (6.5)
ECOG performance status, *n* (%)	
0	45 (36.6)
1	71 (57.7)
2	7 (5.7)
Type of myeloma, *n* (%)	
IgG	65 (52.8)
Non-IgG	21 (17.1)
IgA	20 (16.3)
IgD	1 (0.8)
Light chain	24 (19.5)
Unknown	13 (10.6)
R-ISS disease stage, *n* (%)	
I	28 (22.8)
II	68 (55.3)
III	19 (15.4)
Unknown	8 (6.5)
Cytogenetic risk, *n* (%)	
Standard	83 (67.5)
High^b^	31 (25.2)
Missing	9 (7.3)
Extramedullary disease by BICR, *n* (%)^c^	39 (31.7)
Bone marrow plasma cells, *n* (%)	
<50%	89 (72.4)
≥50%	26 (21.1)
Missing	8 (6.5)
≥1 poor prognosis feature^d^	94 (76.4)
Median no. of prior antimyeloma lines of therapy (range)	5 (2–22)
Prior stem cell transplant, *n* (%)	87 (70.7)
Exposure status, n (%)	
Triple-class^e^	123 (100)
Penta-drug^f^	87 (70.7)
Refractory status, *n* (%)	
Triple-class^e^	119 (96.7)
Penta-drug^f^	52 (42.3)
Refractory to last line of therapy, *n* (%)	118 (95.9)

^a^Includes patients recruited in countries where the collection of races is prohibited.

^b^Includes t(4;14), t(14;16) and del(17p) chromosomal abnormalities.

^c^Extramedullary disease was defined as the presence of any plasmacytoma (extramedullary and/or paramedullary with a soft-tissue component).

^d^Poor prognosis feature refers to at least one of the following: ECOG performance status of 2, R-ISS stage III, high cytogenetic risk, extramedullary disease at baseline, bone marrow plasma cells ≥50% or penta-refractory disease.

^e^Triple-class refers to at least one proteasome inhibitor, one immunomodulatory drug and one anti-CD38 antibody.

^f^Penta-drug refers to at least two proteasome inhibitors, two immunomodulatory drugs and one anti-CD38 antibody.

As of March 14, 2023, 33.3% of patients were still receiving elranatamab (Fig. 1). The median duration of treatment was 5.6 months (range: 0.03–24.4 months), 48.0% were treated for at least 6 months and 35.8% for at least 12 months. The median relative dose intensity for all treatment cycles was 78.4% (range: 8.9–101.3%). The most common primary reasons for permanent treatment discontinuation were progressive disease (PD)/lack of efficacy (41.5%) and AEs (13.8%).

### Primary and secondary efficacy endpoints

**Fig. 2:**
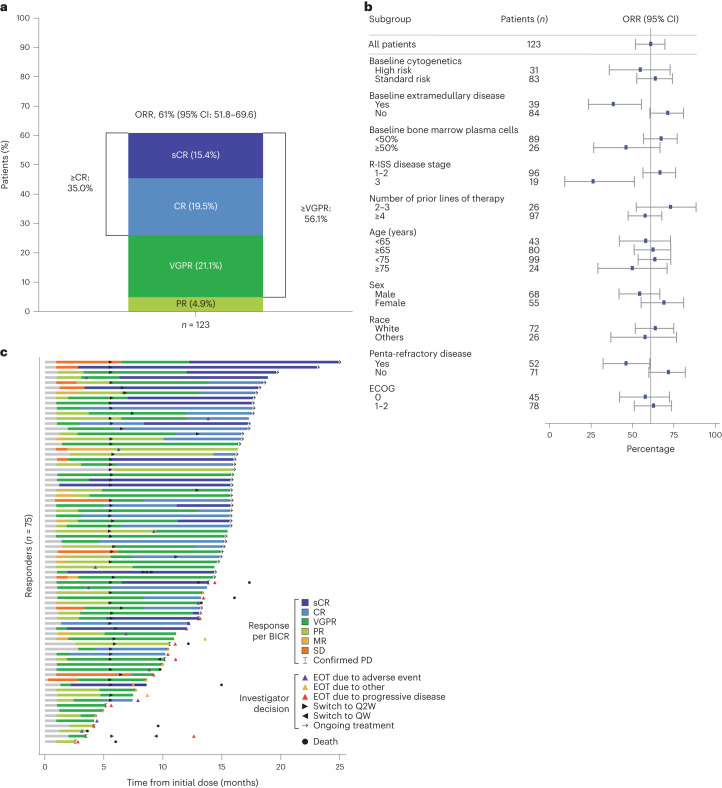
Response to elranatamab in patients with relapsed or refractory multiple myeloma. **a**, Stacked bar graph illustrating the rate of sCR, CR, VGPR and PR in 123 patients who were treated with elranatamab. Responses were assessed by BICR. **b**, Forest plot illustrating the ORR by BICR in subgroups. Blue squares denote ORR, and whiskers indicate 95% CIs. **c**, Swimmer plot showing responses over time in 75 patients who had a response following elranatamab treatment. Responses were assessed by BICR, whereas treatment decisions, including switch to Q2W dosing, were made by the investigator. EOT, end of treatment; MR, minimal response; sCR, stringent complete response; SD, stable disease; VGPR, very good partial response.

**Extended Data Fig. 1:**
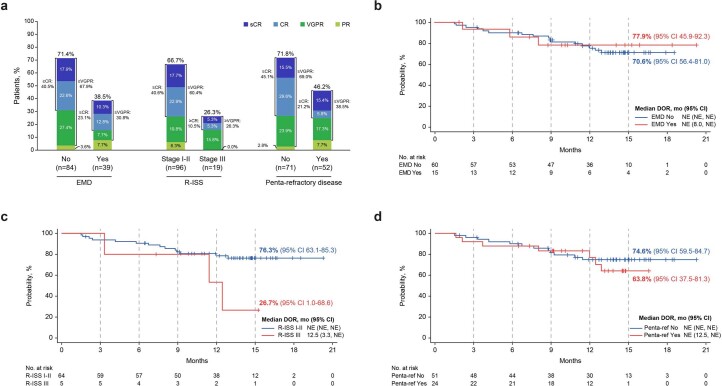
Objective response rate and duration of response (DOR) by blinded independent central review (BICR) in select poor prognosis subgroups. **a**, Stacked bar graphs illustrating the rate of stringent complete response (sCR), complete response (CR), very good partial response (VGPR), and partial response (PR) in patients with or without extramedullary disease (EMD) at baseline, with R-ISS disease stage I-II or III, and with or without penta-refractory disease. Responses were assessed by BICR. **b**, Kaplan-Meier analysis of the DOR in patients with or without EMD. **c**, Kaplan-Meier analysis of the DOR in patients with R-ISS disease stage I-II or III. **d**, Kaplan-Meier analysis of the DOR in patients with or without penta-refractory disease. NE, not estimable; penta-ref, penta-refractory; R-ISS, Revised International Staging System.

After a median follow-up of 14.7 months (range: 0.2–25.1 months), the primary endpoint was met with 61.0% (95% confidence interval (CI): 51.8–69.6) of patients having a confirmed objective response per BICR. The best overall response is summarized in Fig. 2a. A CR or better (≥CR) was achieved in 35.0% of patients, and a very good partial response (VGPR) or better was achieved in 56.1%. At the time of this analysis, 9 (7.3%) responders were still on treatment and had not achieved a CR. MRD negativity was achieved in 89.7% of patients with ≥CR and who were evaluable for MRD (*n* = 29), corresponding to 60.5% of patients with ≥CR. ORRs were higher in patients with R-ISS stage I–II disease and in those without extramedullary disease or penta-refractory disease (Extended Data Fig. 1a). Otherwise, response rates were consistent across subgroups, including in patients with at least 50% bone marrow plasma cells at baseline, and high-risk cytogenetics (Fig. 2b).

**Fig. 3:**
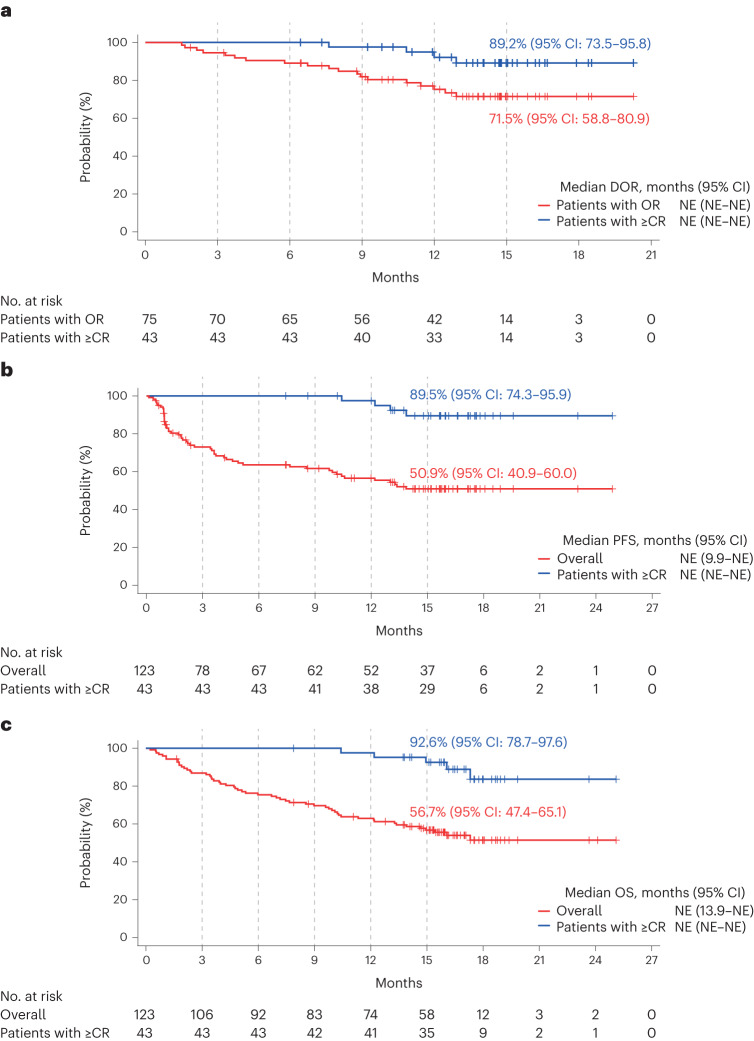
Kaplan–Meier analysis of DOR, PFS and OS. **a**, DOR in 75 patients who had an objective response (OR; red line) and in 43 patients who had CR or better (≥CR) (blue line). **b**, PFS in the overall population (red line) and in 43 patients who had ≥CR (blue line). **c**, OS in the overall population (red line) and in 43 patients who had ≥CR (blue line). Tick marks indicate censored data. NE, not estimable.

In responders, the median TTR was 1.2 months (range: 0.9–7.4 months). Responses deepened over time (Fig. 2c). Among responders, the median DOR was not reached (95% CI: not estimable), with 56 (74.7%) patients censored at the time of analysis. The Kaplan–Meier probability of maintaining the response at 15 months was 71.5% (95% CI: 58.8–80.9) in the overall population and 89.2% (95% CI: 73.5–95.8) in patients with ≥CR (Fig. 3a). The median time to ≥CR was 6.1 months (range: 1.2–14.3 months). In patients with ≥CR, the median DOCR was not reached (95% CI: not estimable) and the probability of maintaining ≥CR at 9 months was 89.0% (95% CI: 69.6–96.4).

Among responders in poor prognosis subgroups (extramedullary disease, penta-refractory disease and R-ISS stage III), the probability of maintaining the response at 15 months was 77.9% (95% CI: 45.9–92.3) versus 70.6% (95% CI: 56.4–81.0) in patients with and without extramedullary disease, respectively; 63.8% (95% CI: 37.5–81.3) versus 74.6% (95% CI: 59.5–84.7) in patients with and without penta-refractory disease, respectively and 76.3% (95% CI: 63.1–85.3) versus 26.7% (95% CI: 1.0–68.6) in patients with R-ISS stages I–II and III disease, respectively (Extended Data Fig. 1b–d).

The median PFS was not reached (95% CI: 9.9 months to not estimable), with 70 (56.9%) patients censored at data cutoff, and the Kaplan–Meier estimate of PFS at 15 months was 50.9% (95% CI: 40.9–60.0; Fig. 3b). The median duration of OS was not reached (95% CI: 13.9 months to not estimable), and the Kaplan–Meier estimate at 15 months was 56.7% (95% CI: 47.4–65.1; Fig. 3c). For patients in ≥CR, the Kaplan–Meier estimates of PFS and OS at 15 months were 89.5% (95% CI: 74.3–95.9) and 92.6% (95% CI: 78.7–97.6), respectively (Fig. 3b,c).

Responses were consistent across BICR, investigator and a computerized algorithm, with an ORR of 61.0% (95% CI: 51.8–69.6), 59.3% (95% CI: 50.1–68.1) and 59.3% (95% CI: 50.1–68.1), respectively. Time-to-event endpoints such as TTR, DOR, DOCR and PFS were also consistent between BICR and investigator (Extended Data Table 1).

### Safety

Treatment-emergent AEs (TEAEs) were reported in all 123 patients treated with elranatamab, with grade 3 or 4 events reported in 87 (70.7%) patients. The most common TEAEs are shown in Table 2. TEAEs led to dose reductions and interruptions in 28.5% and 77.2% of patients, respectively. Hematologic TEAEs (17.1%), including neutropenia (15.4%), were the most frequent (≥15%) TEAEs leading to dose reduction. The most frequent (≥20%) TEAEs leading to dose interruptions were infections (50.4%), most commonly coronavirus disease 2019 (COVID-19) related (25.2%), and hematologic TEAEs (40.7%), most commonly neutropenia (35.0%). Among patients with dose interruptions due to infections or hematologic TEAEs who were rechallenged, 93.5% and 95.3% had a successful rechallenge (defined as able to resume treatment following a dose interruption due to an AE and not discontinued permanently due to the same AE type).

**Table 2:** Treatment-emergent adverse events occurring in ≥20% of patients receiving elranatamab

Treatment-emergent adverse events, *n* (%)	*n* = 123	
	Any grade	Grade 3 or 4
Any treatment-emergent adverse event	123 (100)	87 (70.7)
Hematologic^a^		
Anemia	60 (48.8)	46 (37.4)
Neutropenia	60 (48.8)	60 (48.8)
Thrombocytopenia	38 (30.9)	29 (23.6)
Lymphopenia	33 (26.8)	31 (25.2)
Nonhematologic		
Cytokine release syndrome	71 (57.7)	0
Diarrhea	52 (42.3)	2 (1.6)
Fatigue	45 (36.6)	4 (3.3)
Decreased appetite	41 (33.3)	1 (0.8)
Pyrexia	37 (30.1)	5 (4.1)
COVID-19 related^b^	36 (29.3)^c^	19 (15.4)
Injection site reaction	33 (26.8)	0
Nausea	33 (26.8)	0
Hypokalemia	32 (26.0)	13 (10.6)
Cough	31 (25.2)	0
Headache	29 (23.6)	0

^a^Preferred terms included in hematologic treatment-emergent adverse events are provided in Supplementary Table 2.

^b^Includes preferred terms in COVID-19 (narrow) standardized MedDRA queries.

^c^25/36 (69.4%) patients developed COVID-19 or COVID-19 pneumonia and 10/36 (30.6%) only had a positive SARS-CoV-2 test without developing the disease.

MedDRA, Medical Dictionary for Regulatory Activities.

Infections occurred in 69.9% of patients; 39.8% had grade 3 or 4 events and 6.5% had fatal infections. The most frequently reported were coronavirus disease of 2019 (COVID-19)-related (29.3%; Extended Data Table 2). Among patients with quantitative immunoglobulin data (*n* = 72 at baseline and *n* = 102 postbaseline), 98.6% had immune paresis (defined as at least two uninvolved immunoglobulin isotypes below the lower limit of normal) at baseline and 75.5% had immunoglobulin G (IgG) < 400 mg dl^−1^ at least once during the treatment period. Overall, 43.1% of patients received immunoglobulin replacement during the study. Patients also received anti-infectious prophylaxis per local standard of care. The vast majority of patients (87.0%) received antiviral prophylaxis and approximately half (49.6%) received anti-*Pneumocystis jirovecii* prophylaxis. Few patients received antifungal (11.4%) and antibacterial prophylaxis (5.7%; Extended Data Table 3). Among the six patients who developed *P. jirovecii* pneumonia, only one was receiving prophylaxis.

Peripheral neuropathy, defined as motor dysfunction and sensory neuropathy, was reported in 17.1% and 13.8% of patients, respectively. Of these patients, 14.1% and 35.3% had a history of motor dysfunction and sensory neuropathy, respectively. The most common (≥5%) neuropathic events were muscle spasms and peripheral sensory neuropathy (7.3% each). There were 1 (0.8%) and 0 grade 3 cases of motor dysfunction and sensory neuropathy, respectively, and no grade 4 or 5 events.

**Fig. 4:**
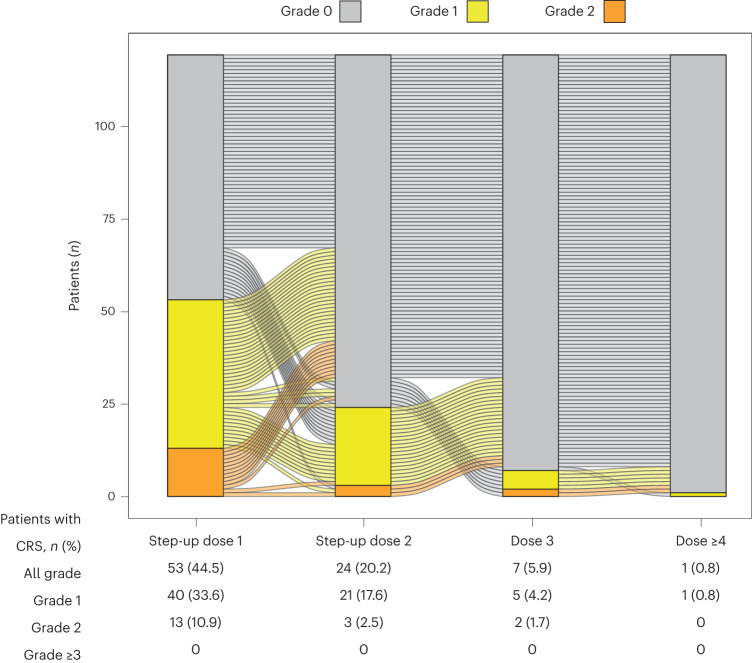
CRS profile of patients who received the 12/32-mg two-step-up priming regimen. CRS experienced by each of the 119 patients who received the 12/32-mg two step-up priming regimen is shown by grade after each dose received. Grade 0 denotes no CRS.

Of the 119 patients who received the two step-up priming dose regimen, cytokine release syndrome (CRS) occurred in 56.3% of patients. All CRS events were grade 1 (42.0%) or grade 2 (14.3%), and no grade 3 or higher events were reported. The median time to onset of CRS relative to the most recent dose was 2.0 d (range: 1.0–9.0 d), and the median time to resolution was 2.0 d (range: 1.0–19.0 d). Overall, 98.8% of CRS events occurred with the first three doses and 90.6% occurred with the step-up doses. One (0.8%) patient had a grade 1 CRS event after the fourth or later dose of elranatamab (Fig. 4). Eighteen (15.1%) patients had more than one CRS event. Tocilizumab and corticosteroids were administered for the treatment of CRS in 22.7% and 8.4% of patients, respectively. ICANS occurred in 4 of 119 (3.4%) patients, with all events grade 1 or 2 (Extended Data Table 4). Supportive treatments for ICANS included corticosteroids (1.7%), tocilizumab (1.7%) and levetiracetam for seizure prophylaxis (0.8%). No patients permanently discontinued elranatamab due to the development of CRS or ICANS.

A total of 55 (44.7%) patients died while on study, the majority (*n* = 37 (30.1%)) due to disease progression. There were 14 (11.4%) patients who died due to non-PD TEAEs, and 8 (6.5%) were due to infections. Four deaths were considered related to elranatamab by the investigator—one patient with adenoviral hepatitis, one with adenovirus infection and pneumonia adenoviral, one with pneumonia pseudomonal and one with failure to thrive. Screening for adenovirus infection was not required per protocol. Treatment recommendations for adenoviral infection were not specified in the protocol, and both patients with grade 5 adenovirus infection were treated by the investigator and received supportive care according to local clinical practice.

### Efficacy and safety with Q2W dosing

**Extended Data Fig. 2:**
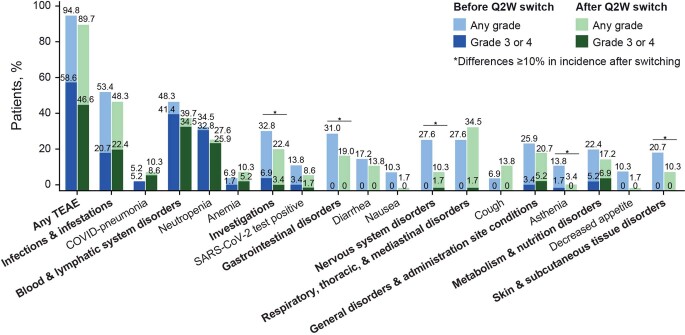
Treatment-emergent adverse events (TEAEs) up to 3 months before and after switching to once every 2 weeks (Q2W) dosing. TEAEs occurring in ≥20% of patients at the level of SOC and in ≥10% of patients at the level of PT in up to 3 months before or after switching to Q2W dosing are reported in the 58 patients who switched to Q2W dosing. Asterisks (*) indicate a difference ≥10% in TEAE incidence after switching to Q2W dosing. COVID, coronavirus disease; PT, preferred terms; SARS-CoV-2, severe acute respiratory syndrome coronavirus 2; SOC, system organ class.

Among responders per BICR who switched to Q2W dosing at least 6 months before the data cutoff date (*n* = 50), 80.0% maintained or improved their response at least 6 months after the switch, with deepening of response observed in 40.0% of patients, including 38.0% who improved their response to ≥CR (Fig. 1c). Of the remaining 20.0%, 2 (4.0%) had confirmed PD, 3 (6.0%) died and 5 (10.0%) permanently discontinued elranatamab while in response. Of all 58 patients who switched to Q2W dosing, the incidence of grade 3 or 4 AEs decreased from 58.6% to 46.6%. The incidence and severity of TEAEs up to 3 months before and after switching to Q2W dosing are presented in Extended Data Fig. 2.

## Discussion

In this phase 2 study in patients with relapsed or refractory multiple myeloma, subcutaneous elranatamab at a dose of 76 mg weekly following a two step-up priming dose regimen of 12 mg and 32 mg during the first week of treatment induced early, deep and durable responses with a manageable safety profile^12–14^. Despite the heavily pretreated population and a high proportion of patients with poor prognostic features at baseline, objective responses occurred in 61.0% of patients, with a majority of responders achieving deep responses; 35% of patients achieved ≥CR, among these, 60.5% were MRD-negative. With a median of follow-up 14.7 months, median DOR, PFS and OS had not been reached. Among responders per BICR who switched from once every week (QW) to Q2W dosing, 80% of patients were still in response at least 6 months after the switch, with responses deepening after the switch suggesting CR may still be achieved with a reduced dose intensity.

A consistent benefit was observed across clinically relevant subgroups, including in patients with an ECOG performance status of 1 or 2, at least 50% bone marrow plasma cells at baseline and high-risk cytogenetics. Although lower ORRs were observed in patients with extramedullary disease at baseline, R-ISS stage III disease and penta-refractory disease, the response rate in these subgroups was clinically meaningful in these typically poor prognosis subgroups. In responders with extramedullary disease and penta-refractory disease, the benefit of elranatamab was maintained over time as DOR was generally consistent compared to the corresponding better prognosis subgroup. The lower response rate observed in patients with extramedullary disease reflects the historically poor prognosis in this subgroup and has also been observed with other anti-BCMA-targeted agents such as belantamab mafodotin and teclistamab^4,19^. Similarly, patients with advanced disease stage (R-ISS stage III) have consistently shown poorer outcomes irrespective of the therapy received^4–6,19–22^. This analysis also showed that patients less heavily pretreated and with less refractory disease had improved response rates, suggesting an increased benefit of elranatamab in earlier treatment settings.

The results of this study are consistent with results reported from the phase 1 MagnetisMM-1 study in which a response rate of 64% and a median DOR of 17.1 months were observed in patients receiving elranatamab at the efficacious dose range (≥215 to 1,000 µg kg^−1^). With the limitation of cross-trial comparisons, the response rate and DOR in patients with relapsed or refractory multiple myeloma treated with elranatamab in MagnetisMM-3 (ORR 61.0%, DOR rate at 12 months 75.3% and PFS at 12 months 56.6%) were comparable to that observed with the recently approved BCMA bispecific antibody, teclistamab, after a median follow-up of 14.1 months (ORR 63.0%, DOR rate at 12 months 68.5% and PFS at 12 months 48.3%), and favorable to the BCMA-targeting antibody–drug conjugate, belantamab mafodotin (ORR 32%, median DOR 11.0 months and median PFS 2.8 months) after a median follow-up of 12.4 months^4,9,19,23^. High response rates have been reported with the BCMA chimeric antigen receptor (CAR) T-cell therapies, ide-cel and cilta-cel, with responses reported in 73% and 97% of treated patients, respectively. Median DOR and PFS were 10.7 months and 8.8 months, respectively, for ide-cel, and were both not reached for cilta-cel^5,6^. However, access to limited, specialized centers able to provide these treatments and/or delayed manufacturing remains a challenge, leaving patients with refractory and rapidly progressing diseases with limited treatment options^7,8^. Similarly, to what has been observed in other studies, the depth of response was associated with improved outcomes, with patients in ≥CR having longer DOR, PFS and OS. Although follow-up is still ongoing, among patients with ≥CR, DOR and PFS at 15 months with elranatamab were comparable to those observed with other BCMA-targeted T-cell-redirecting therapies, suggesting similar outcomes once a deep response is achieved^5,24,25^. Results in this study are also consistent with those observed with bispecific antibodies against other targets such as talquetamab, a bispecific antibody against CD3 and G-protein-coupled receptor, class C, group 5, member D (GPRC5D) (ref. ^26^). A comprehensive analysis with extended follow-up including updated DOR, PFS and OS estimates will be conducted and reported in a future publication.

The most common TEAEs reported in MagnetisMM-3 were CRS, hematologic-related events and infections. Patients who switched to Q2W dosing experienced fewer grade 3 or 4 TEAEs compared to the same time period before switching. However, the interpretation of these findings is limited as no QW comparator group is available to understand the change in AE incidence over time.

With premedication and a two step-up priming dose regimen during the first week of treatment, CRS occurred in 56.3% of patients. All CRS events were grade 1 or 2, with no events grade 3 or higher. CRS events generally occurred early in treatment, with 90.6% limited to the step-up doses. Only one patient had a CRS event after the fourth or later dose, and few patients had more than one CRS event. ICANS was infrequent (occurring in 3.4% of patients) and limited to grade 1 or 2 events. Although the study protocol required hospitalization for the step-up priming doses (48 h after the first dose, 24 h after the second dose), the predictable and manageable profile of CRS and ICANS supports the potential for outpatient administration.

Although the toxicities associated with BCMA-targeted T-cell-redirecting therapies are similar due to their mechanisms of action, the frequency and severity may vary between agents. CRS is the most frequent AE observed with T-cell-redirecting therapies; however, the incidence and severity are lower with bispecific antibodies^7–9,27^. While cross-trial comparisons should be interpreted with caution, elranatamab administered with a two step-up priming regimen and premedication showed a lower CRS incidence (56.3% versus 72.1%), as well as fewer repeat CRS events (15.1% versus 33.3%) compared to teclistamab. Higher rates of CRS (85–95%, including grade 5 events) have been reported in studies of BCMA-directed CAR T cells. Similarly, neurotoxicity including ICANS has been observed more frequently with BCMA-directed CAR T cells than with bispecific antibodies^7–9,27^.

Peripheral neuropathy is a common complication of multiple myeloma and its treatment, especially with early-generation proteasome inhibitors and immunomodulatory drugs such as bortezomib and thalidomide, respectively^28,29^. More recently, neuropathy has also been observed with BCMA-targeted T-cell-redirecting therapies such as CAR T cells and bispecific antibodies^7–9^. Although cross-study comparisons are limited due to the different definitions of neuropathy across studies, the incidences seem comparable across T-cell-redirecting therapies with rates of motor dysfunction and sensory neuropathy of 16% and 15%, respectively, with teclistamab, 11% and 17% of motor dysfunction and neuropathy peripheral, respectively, with ide-cel and 16% motor dysfunction with cilta-cel. In this study, the incidence of motor dysfunction and sensory neuropathy was 17.1% and 13.8%, respectively. No Parkinson-like or fatal neuropathy events have been observed with elranatamab.

Hematologic AEs were frequent, with the most common AEs being neutropenia and anemia. In comparison to other BCMA-targeted T-cell-redirecting therapies, the rate of hematologic AEs with elranatamab was similar or lower after a similar follow-up^4–6^. The incidence of grade 3 or 4 thrombocytopenia was similar to that observed with teclistamab despite the lower inclusion threshold (≥25 × 10^9^ l^−1^) in this study. Hematologic AEs were generally manageable with dose reductions and/or interruptions, as well as with supportive therapies. The majority of patients were able to continue treatment with elranatamab.

BCMA-targeted T-cell-redirecting therapies have been linked to a heightened susceptibility to infectious complications due to their mechanism of action^30–33^. Inhibiting BCMA signaling, which is critical for the survival and proliferation of plasma cells, may worsen the pre-existing myeloma-induced immunosupression^31,32,34^. In this study, among patients with quantitative immunoglobulin data, almost all had immune paresis at baseline and a substantial proportion had IgG < 400 mg dl^−1^ while on treatment. The most frequently reported infectious disease was COVID-19, coinciding with the ongoing pandemic during the study period. Following emerging data on the infection risk profile observed during the conduct of this study and reported with other BCMA T-cell-redirecting therapies,^4–6,35^ recommendations for infection prophylaxis were added to this study as a protocol amendment in July 2022. Before this protocol amendment, antimicrobial prophylaxis was used at the investigator’s discretion. Close monitoring to ensure early recognition of infection, as well as the institution of antimicrobial prophylaxis and immunoglobulin replacement therapy, should be a priority for patients with multiple myeloma receiving BCMA-targeted T-cell-redirecting therapies^36^. Recently published consensus guidelines provide additional information on how to manage infections in patients with multiple myeloma receiving BCMA-directed therapies^36–39^. T-cell-redirecting therapies against other targets (for example, GPRC5D) are also under development. While CRS and ICANS are associated with T-cell activation and occur irrespective of the target, other target-specific toxicities vary across treatments. For example, anti-GPRC5D-targeted agents have been associated with specific toxicities such as dysgeusia and skin and nail toxicity^26^. The selection of targeted therapies for patients with relapsed or refractory multiple myeloma following treatment with immunomodulatory drugs, proteasome inhibitors and anti-CD38 antibodies will depend on disease characteristics and patient-related factors such as comorbidities and toxicities with prior treatments.

The interpretation of the results in this study is limited by its single-arm design and lack of direct comparison with other treatment options, as well as by the small sample size in some subgroups. Longer-term follow-up is required to confirm benefits in DOR, PFS and OS. However, current results support further investigation of elranatamab. An ongoing open-label, randomized phase 3 study is evaluating elranatamab monotherapy versus elranatamab + daratumumab versus standard of care daratumumab + pomalidomide + dexamethasone in patients with relapsed or refractory multiple myeloma who have received lenalidomide and a proteasome inhibitor (NCT05020236).

In this phase 2 study in heavily pretreated patients with relapsed or refractory multiple myeloma, elranatamab demonstrated a high rate of deep and durable responses, including in patients achieving ≥CR, with a manageable safety profile. Administration of a two step-up priming dose regimen successfully mitigated the rate and severity of CRS with a predictable profile supporting the potential for outpatient administration. Although additional follow-up is needed, maintenance or deepening of response was observed with elranatamab following the switch to a biweekly schedule. Biweekly administration may provide greater patient convenience with potentially less toxicity. Elranatamab is also a readily accessible, off-the-shelf therapy, which provides an option for patients unable to access CAR T-cell therapy. These results support the continued development of elranatamab as monotherapy and its further investigation in combination with standard or new therapies for patients with multiple myeloma.

## Methods

### Study design and patients

MagnetisMM-3 is an ongoing, multicenter, open-label, single-arm, phase 2 study to evaluate the efficacy and safety of elranatamab monotherapy in patients with relapsed or refractory multiple myeloma.

Eligible patients were male or female (if not pregnant or breastfeeding), 18 years of age or older, willing to follow protocol-specified requirements and complete scheduled visits, with a prior diagnosis of multiple myeloma and measurable disease as defined by IMWG criteria^17^. Patients had to have a disease that was refractory (defined as having disease progression while on therapy, or within 60 d of the last dose in any line, regardless of response) to at least one proteasome inhibitor, one immunomodulatory drug and one anti-CD38 antibody, and had to be relapsed or refractory to their last antimyeloma regimen. Patients eligible for cohort A must not have received prior BCMA-directed therapy, while patients eligible for Cohort B must have received prior BCMA-directed antibody–drug conjugate or BCMA-directed CAR T-cell therapy, either approved or investigational. Patients were required to have an ECOG performance status ≤2, adequate bone marrow function (characterized by platelets ≥25 × 10^9^ l^−1^, absolute neutrophil count ≥1.0 × 10^9^ l^−1^ and hemoglobin ≥8 g dl^−1^), adequate hepatic function (defined as total bilirubin ≤2× ULN (≤3× ULN if documented Gilbert’s syndrome), aspartate aminotransferase ≤2.5× ULN and alanine aminotransferase ≤2.5× ULN), adequate renal function (defined as estimated creatinine clearance ≥30 ml min^−1^) and left ventricular ejection fraction ≥40%. Acute effects of any prior therapy must have resolved to baseline severity or NCI CTCAE grade ≤1.

Patients were excluded if they had smoldering multiple myeloma, active plasma cell leukemia, amyloidosis or polyneuropathy, organomegaly, endocrinopathy, monoclonal plasma cell disorder, skin changes syndrome (POEMS), a stem cell transplant ≤12 weeks before enrollment or active graft versus host disease, or any active, uncontrolled bacterial, fungal or viral infection (including active hepatitis B virus, hepatitis C virus, SARS-CoV-2 or human immunodeficiency virus). Active infections had to be resolved at least 14 d before enrollment. Patients were also excluded if they had impaired cardiovascular function or clinically meaningful cardiovascular disease (defined as acute myocardial infarction, acute coronary syndromes, clinically meaningful cardiac arrhythmias, thromboembolic or cerebrovascular events or prolonged QT syndrome) ≤6 months before enrollment, ongoing grade ≥2 peripheral sensory or motor neuropathy, history of Guillain–Barré syndrome or variants or history of any grade ≥3 peripheral motor polyneuropathy, or for cohort B, history of any grade peripheral sensory or motor neuropathy with prior BCMA-directed therapy. Patients with another active malignancy within 3 years before enrollment (except for adequately treated basal cell or squamous cell skin cancer or carcinoma in situ), known or suspected hypersensitivity to elranatamab, previous administration of an investigational drug within 30 d or five half-lives preceding the first dose of elranatamab (whichever was longer), previous treatment with an anti-BCMA bispecific antibody or who received a live attenuated vaccine within 4 weeks of the first dose of treatment were also excluded. Patients were also ineligible if they had surgical, medical or psychiatric conditions or laboratory abnormalities that may increase the risk of study participation or (per investigator’s judgment) make the patient inappropriate for the study.

Patients were assigned to 1 of 2 independent, parallel cohorts. Efficacy and safety results in patients naïve to BCMA-directed therapies are reported here (Fig. 1). Results from patients with prior BCMA-directed therapy at baseline will be reported separately.

### Study oversight

The study was designed by the authors in conjunction with the sponsor and conducted in accordance with the principles of the Declaration of Helsinki and the International Council for Harmonisation Guidelines for Good Clinical Practice. The study protocol and amendments were approved by the institutional review boards at participating sites. The study used an external data monitoring committee, which was responsible for ongoing safety monitoring during the study conduct as well as the prespecified interim futility and efficacy analyses. All patients provided written informed consent.

### Treatment

All patients received subcutaneous elranatamab 76 mg QW on a 28-d cycle with a two step-up priming dose regimen of 12 mg on day 1 and 32 mg on day 4 during the first week, with the exception of the first four patients enrolled in the study who received a single priming dose of 44 mg on day 1 before receiving the full dose of 76 mg starting on day 8. These first four patients were enrolled before the protocol was amended to include the 12/32 mg step-up regimen. Hospitalization was required for 48 h following the first step-up dose and for 24 h after the second step-up dose. Premedication with acetaminophen (650 mg or equivalent), diphenhydramine (25 mg or equivalent) and dexamethasone (20 mg or equivalent) was required before each step-up dose and before the first full dose of elranatamab. Patients who received QW dosing for at least six cycles and achieved a PR or better (≥PR) persisting for at least 2 months had their dosing interval changed to Q2W. Dose reductions and interruptions were permitted for toxicity. Elranatamab treatment was to be continued until disease progression, unacceptable toxicity or withdrawal of consent.

### Efficacy and safety assessments

The primary endpoint was confirmed ORR, defined as PR or better (≥PR) according to IMWG criteria^17^, as assessed by BICR. Secondary endpoints included ORR by BICR baseline extramedullary disease status, ORR by investigator, CR rate (defined as CR or better (≥CR)), TTR, DOR, DOCR, MRD negativity rate at a sensitivity threshold of 10^−5^ (assessed via next-generation sequencing of DNA from bone marrow aspirates using the clonoSEQ MRD assay from Adaptive Biotechnologies) and PFS per IMWG criteria. Additional secondary endpoints were OS, safety, pharmacokinetics and immunogenicity. The MRD negativity rate was defined as the proportion of patients with ≥CR and with negative MRD from the date of the first dose until confirmed PD, death or start of new anticancer therapy, whichever occurred first. DOR (for patients with confirmed objective responses) was the time from the first confirmed response to confirmed PD or death due to any cause, whichever was earlier, or censoring. PFS was the time from the first dose to confirmed PD or death due to any cause, whichever was earlier, or censoring. For DOR and PFS, patients were censored at the last valid assessment before (1) initiation of new anticancer or (2) two consecutive missed efficacy assessments before an event. OS was the time from the first dose to death due to any cause or censoring. Patients not known to have died were censored at the last contact date. A prespecified sensitivity analysis evaluated response by a computerized algorithm. Responses were derived per IMWG based on the local laboratory and bone marrow data and the individual lesion data provided by the investigator. The impact of switching from QW to Q2W dosing on efficacy was assessed in responders per BICR who had switched to Q2W dosing ≥6 months before the data cutoff date. Patients who were in response by investigator but not by BICR at the time of the switch or who had <6 months of possible follow-up from the time of the switch to the time of data cutoff were excluded. Patients were counted as responders after the switch if they had an assessment demonstrating a response ≥6 months after the switch.

AEs were graded according to the NCI CTCAE (version 5.0), except for CRS and ICANS, which were graded according to the criteria of the American Society for Transplantation and Cellular Therapy^18^. TEAEs were defined as any event occurring from the first dose of elranatamab through the minimum of 90 d after the last elranatamab dose or the start of new anticancer therapy. See Supplementary Table 2 for the list of Medical Dictionary for Regulatory Activities (MedDRA version 25.1) preferred terms included in clustered terms for hematologic and peripheral neuropathy TEAEs. The standardized MedDRA query COVID-19 (narrow) was used for COVID-19 related TEAEs. The impact of switching from QW to Q2W dosing on safety was assessed by comparing the incidence of TEAEs before and after the switch. New-onset TEAEs (including those which increased in grade) for each patient were included for an equal time period before and after the switch (based on individual patient follow-up time after the switch), with a maximum time period of up to 3 months.

All analyses were performed in the 123 patients who received at least one dose of elranatamab, with the exception of CRS and ICANS analyses, which were performed on the 119 patients who received the 12/32 mg step-up regimen.

### Statistical analysis

Efficacy and safety were evaluated in all patients enrolled who received at least one dose of elranatamab (safety analysis set). A sample size of 120 patients was estimated to give a power of at least 98% to establish an ORR of more than 30% at a one-sided significance level of 0.025, assuming an ORR of at least 48%. As specified in the protocol and statistical analysis plan, if the null hypothesis for ORR by BICR (defined as ≤30% by IMWG) was rejected for cohort A, the key secondary endpoint of ORR by BICR for those without EMD at baseline was tested in a hierarchical fashion using the gatekeeping procedure that the ORR is ≤38% with a one-sided significance level of 0.025. If the null hypothesis for ORR by BICR for those without EMD at baseline was rejected for cohort A, the key secondary endpoint of ORR by BICR for those with EMD at baseline was tested in a hierarchical fashion using the gatekeeping procedure that the ORR is ≤12% with a one-sided significance level of 0.025. No other adjustments for multiple comparisons were made. Descriptive statistics were used for efficacy and safety outcomes unless otherwise stated. Exact two-sided 95% CIs were included for response endpoints. Time-to-event endpoints—except TTR—were summarized using the Kaplan–Meier method. Medians, rates at 15 months and their two-sided 95% CIs were included. The CIs for the median were calculated according to Brookmeyer and Crowley, and the CIs for the survival function estimates at particular time points were derived using the log(−log) method. Data cutoff for efficacy and safety was March 14, 2023, except for CRS and ICANS data, which was based on January 12, 2023.

### Reporting summary

Further information on research design is available in the Nature Portfolio Reporting Summary linked to this article.

## Online content

Any methods, additional references, Nature Portfolio reporting summaries, source data, extended data, supplementary information, acknowledgements, peer review information; details of author contributions and competing interests; and statements of data and code availability are available at 10.1038/s41591-023-02528-9.

## Supplementary information

Supplementary Information Supplementary Tables 1 and 2.

Reporting Summary

## Figures and Tables

**Extended Data Table 1:**
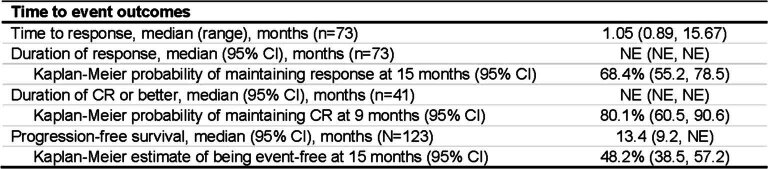
Time to event outcomes by investigator per International Myeloma Working Group criteria in patients receiving elranatamab

CR, complete response; NE, not estimable.

**Extended Data Table 2:**
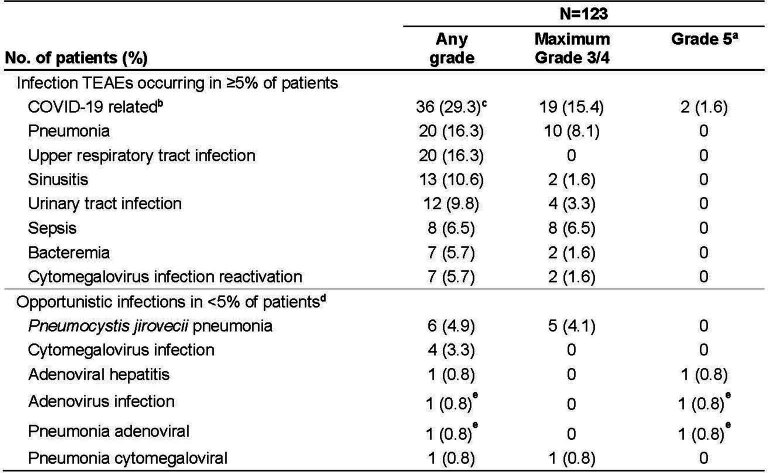
Infection treatment-emergent adverse events in patients receiving elranatamab

COVID-19, coronavirus disease 2019; MedDRA Medical Dictionary for Regulatory Activities; TEAE, treatment-emergent adverse event.

^a^3 (2.4%) patients had grade 5 septic shock.

^b^Includes preferred terms in COVID-19 (narrow) standardized MedDRA queries.

^c^25/36 (69.4%) patients developed COVID-19 or COVID-19 pneumonia and 10/36 (30.6%) only had a positive SARS-CoV-2 test without developing the disease.

^d^Opportunistic infection TEAEs includes preferred terms: adenoviral hepatitis, adenovirus infection, cytomegalovirus infection, cytomegalovirus infection reactivation, cytomegalovirus viremia, pneumonia adenoviral, pneumonia cytomegaloviral, *Pneumocystis jirovecii* pneumonia.

^e^Preferred terms both reported in the same patient.

**Extended Data Table 3:**
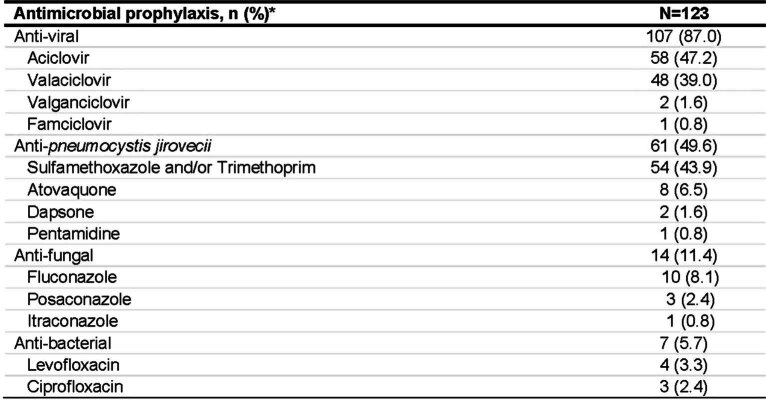
Antimicrobial agents administered for infection prophylaxis

*Criteria for distinguishing antimicrobial agent administration for prophylaxis vs other indication (eg, treatment of active infection) included continuous treatment for at least 14 days and not administered to treat an adverse event.

**Extended Data Table 4:**
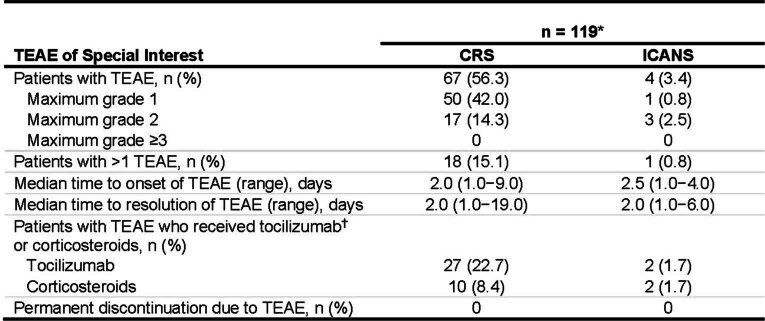
Characteristics and management of cytokine release syndrome and immune effector cell-associated neurotoxicity syndrome in patients who received the two step-up priming regimen

CRS, cytokine release syndrome; ICANS, immune effector cell-associated neurotoxicity syndrome; TEAE, treatment-emergent adverse event.

*The 4 patients who did not receive the two step-up priming regimen were excluded from this CRS and ICANS analysis.

^s^Includes tocilizumab and siltuximab.

## Data Availability

Upon request, and subject to review, Pfizer will provide the data that support the findings of this study. Subject to certain criteria, conditions and exceptions, Pfizer may also provide access to the related individual de-identified patient data. See https://www.pfizer.com/science/clinical-trials/trial-data-and-results for more information. The study protocol and statistical analysis plan for MagnetisMM-3 have been uploaded to clinicaltrial.gov.
