# Supplementary material for: Elranatamab in relapsed or refractory multiple myeloma: phase 2 MagnetisMM-3 trial results
Source: Nat Med. 2023 Aug 15;29(9):2259–67. doi: 10.1038/s41591-023-02528-9 (PMC10504075; doi:10.1038/s41591-023-02528-9)
Supplement: Supplementary file 1 — Supplementary Tables 1 and 2. [file 41591_2023_2528_MOESM1_ESM.pdf]

nature medicine

Article https://doi.org/10.1038/s41591-023-02528-9

Elranatamab in relapsed or refractory multiple myeloma: phase 2 MagnetisMM-3 trial results

In the format provided by the authors and unedited

Supplementary information

Supplementary Table 1. MagnetisMM-3 study investigators.

United States	
Maria Chaudhry	Ruben Niesvizky
Catlin Costello	Ajay Nooka
Andrew Dalovisio	Fahd Quddus
Suzanne R Fanning	Noopur Raje
Afshin Eli Gabayan	Seema B Singhal
Jens Hillengass	Melhem Solh
Yogesh Jethava	Don A Stevens
Guenther Koehne	Patrick J Stiff
Sarah Larson	Michael H Tomasson
Lisa Lee	Asya Nina Varshavsky-Yanovsky
Alexander Lesokhin	David H Vesole
Christopher Maisel	
Canada	
Nizar Bahlis	Richard LeBlanc
Michael Chu	Michael Sebag
Richard Kaedbey	Suzanne M Trudel
Marc Lalancette	
Spain	
Aranzazu Alonso	Maria V Mateos Manteca
Carlos J Fernández de Larrea Rodríguez	Albert Oriol Rocafiguera
Marta S Gonzalez Perez	Maria Ribas García
Joaquin Martinez Lopez	Paula Rodríguez Otero
Japan	
Hiroshi Handa	Shigeki Ito
Shinsuke Iida	Yuya Nagai
Tadao Ishida	Hisayuki Yokoyama
Kenichi Ishizawa	
**France**	
Bertrand Arnulf	Salomon Manier
Lionel Karlin	Mohamad Mohty
Xavier Leleu	Cyrille Touzeau
**Germany**	
Mathias Haenel	Natalie Schub
Stefan Knop	Katja Weisel
Marc-Steffen Raab	
Australia	
Philip J Campbell	Hang Quach
H Miles Prince	Andrew Spencer
Belgium	
Julien Depaus	Ka Lung Wu
Ann Van De Velde	
Poland	
Jaroslaw Czyz	Tomasz Wrobel
Dominik Dytfeld	
United Kingdom	
Karthikeyan Arumugam Ramasamy	Emma Searle
Charlotte Pawlyn	

Supplementary Table 2. List of MedDRA preferred terms included in clustered terms for hematologic and peripheral neuropathy TEAEs.

Clustered term	MedDRA preferred terms included
Hematologic	
Thrombocytopenia	Thrombocytopenia, Platelet count decreased
Anemia	Anemia, Hemoglobin decreased, Red blood cell count decreased, Hematocrit decreased, Normochromic anemia, Normocytic anemia, Normochromic normocytic anemia)
Neutropenia	Neutropenia, Neutrophil count decreased, Neutrophil percentage decreased, Cyclic neutropenia, Agranulocytosis, Granulocytopenia, Granulocyte count decreased
Leukopenia	Leukopenia, White blood cell count decreased
Lymphopenia	Lymphopenia, Lymphocyte count decreased, Lymphocyte percentage decreased, CD4 lymphocytes decreased, CD4 lymphocyte percentage decreased, CD8 lymphocytes decreased, CD8 lymphocyte percentage decreased
Peripheral neuropathy	
Motor dysfunction	Ataxia, Balance disorder, Gait disturbance, Motor dysfunction, Muscle contracture, Muscle spasms, Muscular weakness, Peripheral motor neuropathy, Peroneal nerve palsy, Tremor
Sensory neuropathy	Burning sensation, Dysesthesia, Hypoesthesia, Neuropathy peripheral, Paraesthesia, Parosmia, Peripheral sensorimotor neuropathy, Peripheral sensory neuropathy, Sensory loss, Polyneuropathy

MedDRA Medical Dictionary for Regulatory Activities; TEAE, treatment-emergent adverse event.
