# Supplementary material for: Elranatamab in relapsed or refractory multiple myeloma: phase 2 MagnetisMM-3 trial results
Source: Nat Med. 2023 Aug 15;29(9):2259–67. doi: 10.1038/s41591-023-02528-9 (PMC10504075; doi:10.1038/s41591-023-02528-9)
Supplement: Supplementary file 2 — Reporting Summary [file 41591_2023_2528_MOESM2_ESM.pdf]

nature portfolio

Corresponding author(s): Alexander M. Lesokhin

Last updated by author(s): Jul 28, 2023

Reporting Summary

Nature Portfolio wishes to improve the reproducibility of the work that we publish. This form provides structure for consistency and transparency in reporting. For further information on Nature Portfolio policies, see our Editorial Policies and the Editorial Policy Checklist.

Statistics

For all statistical analyses, confirm that the following items are present in the figure legend, table legend, main text, or Methods section.

n/a	Confirmed	
☐	☒	The exact sample size (*n*) for each experimental group/condition, given as a discrete number and unit of measurement
☒	☐	A statement on whether measurements were taken from distinct samples or whether the same sample was measured repeatedly
☐	☒	The statistical test(s) used AND whether they are one- or two-sided<br>*Only common tests should be described solely by name; describe more complex techniques in the Methods section.*
☒	☐	A description of all covariates tested
☐	☒	A description of any assumptions or corrections, such as tests of normality and adjustment for multiple comparisons
☐	☒	A full description of the statistical parameters including central tendency (e.g. means) or other basic estimates (e.g. regression coefficient) AND variation (e.g. standard deviation) or associated estimates of uncertainty (e.g. confidence intervals)
☐	☒	For null hypothesis testing, the test statistic (e.g. *F*, *t*, *r*) with confidence intervals, effect sizes, degrees of freedom and *P* value noted<br>*Give P values as exact values whenever suitable.*
☒	☐	For Bayesian analysis, information on the choice of priors and Markov chain Monte Carlo settings
☒	☐	For hierarchical and complex designs, identification of the appropriate level for tests and full reporting of outcomes
☒	☐	Estimates of effect sizes (e.g. Cohen's *d*, Pearson's *r*), indicating how they were calculated

Our web collection on statistics for biologists contains articles on many of the points above.

Software and code

Policy information about availability of computer code

Data collection	Pfizer InForm EDC platform
Data analysis	SAS version 9.4 to perform all data analyses. Fig. 4 was produced using R 4.1.0

For manuscripts utilizing custom algorithms or software that are central to the research but not yet described in published literature, software must be made available to editors and reviewers. We strongly encourage code deposition in a community repository (e.g. GitHub). See the Nature Portfolio guidelines for submitting code & software for further information.

Data

Policy information about availability of data

All manuscripts must include a data availability statement. This statement should provide the following information, where applicable:

- Accession codes, unique identifiers, or web links for publicly available datasets
- A description of any restrictions on data availability
- For clinical datasets or third party data, please ensure that the statement adheres to our policy

(Per Pfizer policy, the following standard text is used:) Upon request, and subject to review, Pfizer will provide the data that support the findings of this study. Subject to certain criteria, conditions, and exceptions, Pfizer may also provide access to the related individual de-identified participant data. See https://www.pfizer.com/science/clinical-trials/trial-data-and-results for more information.

Research involving human participants, their data, or biological material

Policy information about studies with human participants or human data. See also policy information about sex, gender (identity/presentation), and sexual orientation and race, ethnicity and racism.

Reporting on sex and gender	Reporting sex per clinical trial protocol
Reporting on race, ethnicity, or other socially relevant groupings	Reporting both race and ethnicity per clinical trial protocol
Population characteristics	Among the 123 patients who received elranatamab, the median age was 68.0 years (range, 36, 89), 55.3% were male, and 58.5% were White, 13.0% Asian, 7.3% Black/African American (Table 1). At baseline, 63.4% patients had an ECOG performance status of 1 or 2, 15.4% had stage III disease according to the Revised International Staging System (R-ISS), and 25.2% had high-risk cytogenetics, defined as t(4;14), t(14;16), or del(17p). Extramedullary disease, defined as the presence of any plasmacytoma (extramedullary and/or paramedullary with a soft-tissue component), assessed by BICR, was present in 31.7% of patients. Overall, 76.4% had at least one poor prognostic feature (Table 1). Patients had received a median of 5 (range, 2, 22) prior lines of therapy, 96.7% had triple-class refractory disease, and 42.3% had penta-drug refractory disease (refractory to at least two proteasome inhibitors, two immunomodulatory drugs, and one anti-CD38 antibody).
Recruitment	MagnetisMM-3 is an ongoing, multicenter, open-label, single-arm, phase 2 study investigating the efficacy and safety of elranatamab in patients with relapsed or refractory multiple myeloma. Eligible patients were 18 years of age or older with a prior diagnosis of multiple myeloma and measurable disease per IMWG criteria, adequate bone marrow (platelets ≥25 x 109/L, absolute neutrophil count ≥1.0 x 109/L, hemoglobin ≥8 g/dL), hepatic (total bilirubin ≤2x upper limit of normal [ULN; ≤3x ULN if documented Gilbert's syndrome], aspartate aminotransferase ≤2.5x ULN, and ≤2.5x ULN alanine aminotransferase), and renal (creatinine clearance ≥30 ml/min) function, and an Eastern Cooperative Oncology Group (ECOG) performance status ≤2. Patients had to have disease refractory to at least 1 proteasome inhibitor, 1 immunomodulatory drug, and 1 anti-CD38 antibody, and disease relapsed or refractory to their last anti-myeloma regimen. Those in Cohort A must not have received prior BCMA-directed therapy. From February 9, 2021 through January 7, 2022, a total of 123 patients were enrolled in Cohort A and dosed at 47 study sites in 10 countries (Fig. 1; Supplementary Table 1).
Ethics oversight	The study was designed by the authors in conjunction with the sponsor and conducted in accordance with the principles of the Declaration of Helsinki and the International Council for Harmonisation guidelines for Good Clinical Practice. The study protocol and amendments were approved by the institutional review boards at participating sites. All patients provided written informed consent.

Note that full information on the approval of the study protocol must also be provided in the manuscript.

Field-specific reporting

Please select the one below that is the best fit for your research. If you are not sure, read the appropriate sections before making your selection.

☒ Life sciences ☐ Behavioural & social sciences ☐ Ecological, evolutionary & environmental sciences

For a reference copy of the document with all sections, see nature.com/documents/nr-reporting-summary-flat.pdf

Life sciences study design

All studies must disclose on these points even when the disclosure is negative.

Sample size	A sample size of 120 patients in Cohort A was estimated to give a power of at least 98% to establish an objective response rate of more than 30% at a one-sided significance level of 0.025, assuming an objective response rate of at least 48%.
Data exclusions	None
Replication	Not applicable because each patient is an individual and different
Randomization	Clinical trial reported in this manuscript is nonrandomized. This is a single-arm phase 2 study
Blinding	Clinical trial reported in this manuscript is not blinded. This phase 2 study is open-label

Reporting for specific materials, systems and methods

We require information from authors about some types of materials, experimental systems and methods used in many studies. Here, indicate whether each material, system or method listed is relevant to your study. If you are not sure if a list item applies to your research, read the appropriate section before selecting a response.

Materials & experimental systems

n/a	Involved in the study
☐	☒ Antibodies
☒	☐ Eukaryotic cell lines
☒	☐ Palaeontology and archaeology
☒	☐ Animals and other organisms
☐	☒ Clinical data
☒	☐ Dual use research of concern
☒	☐ Plants

Methods

n/a	Involved in the study
☒	☐ ChIP-seq
☒	☐ Flow cytometry
☒	☐ MRI-based neuroimaging

Antibodies

Antibodies used

Elranatamab is a proprietary bispecific antibody under development by the clinical study sponsor (Pfizer). No other antibodies were used in the study

Validation

Data provided in this paper validates the use of elranatamab in patients. The targets of elranatamab were validated in preclinical studies and the data are proprietary to the study sponsor (Pfizer Inc)

Clinical data

Policy information about clinical studies

All manuscripts should comply with the ICMJE guidelines for publication of clinical research and a completed CONSORT checklist must be included with all submissions.

Clinical trial registration

NCT04649359

Study protocol

Included with submission. Will be posted on clinicaltrials.gov at a later time

Data collection

From February 9, 2021 through January 7, 2022, a total of 123 patients were enrolled in Cohort A and dosed at 47 study sites in 10 countries (Fig. 1; Supplementary Table 1)

Outcomes

The primary endpoint was objective response rate (ORR) by blinded independent central review (BICR) per International Myeloma Working Group (IMWG) criteria.17 Secondary endpoints included ORR by BICR baseline extramedullary disease status, ORR by investigator, complete response (CR) rate (defined as CR or better), time to response (TTR), duration of response (DOR), duration of CR or better (DOCR), MRD negativity rate, PFS, OS, safety, pharmacokinetics, and immunogenicity
